# The factor structure and construct validity of the inventory of callous-unemotional traits in Chinese undergraduate students

**DOI:** 10.1371/journal.pone.0189003

**Published:** 2017-12-07

**Authors:** Meng-Cheng Wang, Yu Gao, Jiaxin Deng, Hongyu Lai, Qiaowen Deng, Cherie Armour

**Affiliations:** 1 Department of Psychology, Guangzhou University, Guangzhou, China; 2 The Key Laboratory for Juveniles Mental Health and Educational Neuroscience in Guangdong Province, Guangzhou, China; 3 Brooklyn College, the City University of New York, Brooklyn, New York, United States of America; 4 The Graduate Center of the City University of New York, New York, New York, United States of America; 5 Psychology Research Institute, University of Ulster, Coleraine, United Kingdom; Xi'an Jiaotong University School of Medicine, CHINA

## Abstract

The current study assesses the factor structure and construct validity of the self-reported Inventory of Callous–Unemotional Traits (ICU) in 637 Chinese community adults (mean age = 25.98, *SD* = 5.79). A series of theoretical models proposed in previous studies were tested through confirmatory factor analyses. Results indicated that a shortened form that consists of 11 items (ICU-11) to assess callousness and uncaring factors has excellent overall fit. Additionally, correlations with a wide range of external variables demonstrated that this shortened form has similar construct validity compared to the original ICU. In conclusion, our findings suggest that the ICU-11 may be a promising self-report tool that could be a good substitute for the original form to assess callous-uncaring traits in adults.

## Introduction

Psychopathic personality is a multifaceted personality disorder comprised of the interpersonal, affective, and behavioral/lifestyle dimensions[1]. There is growing evidence that the affective component of psychopathy, also called callous and unemotional (CU) traits, could define an important subgroup of children and adolescents with severe conduct problems[2]. CU traits are characterized by a lack of concern about performance, lack of guilt and empathy, and a shallow and deficient affect [3]. These traits are believed to be the developmental precursor to adult psychopathy [3–5]. Increasing understanding of the developmental progression of CU traits from childhood to adulthood has received increased attention [6, 7]. Therefore, the development of a measure that is appropriate to use in various age groups is timely, necessary, and critical. One way to do this is to validate existing youth measurements in adult samples. Until recently, only a few studies have attempted to address this issue [8–11]. For example, in a sample of 687 college students, Kimonis and colleagues found that a three-factor structure similar to that found in youth fit the data well through principal components analyses. The final model also showed reasonable convergent and discriminant validity. Other instruments that were developed initially for youth (i.e., the Youth Psychopathic Traits Inventory) have also been validated in adult populations [10, 11]. Despite those promising results, more validation studies are warranted, and the current study will add to this area of research.

Given the importance of CU traits for understanding antisocial and delinquent youths, there is a need for an efficient, reliable, and valid measure of these traits. The two most widely used measures are the Psychopathy Checklist: Youth Version (PCL: YV)[12] and the Antisocial Process Screening Device (APSD)[13]. The PCL: YV is a 60–90 minute semi-structured interview and has primarily been used in incarcerated samples of adolescents (ages 12 to 18). It is a time-consuming instrument and thus is less appropriate for use in community samples. Furthermore, it contains only a few items that specifically assess CU traits (*n* = 4).

The APSD is a 20-item rating scale including parent, teacher [13], and self-report [14] versions. However, this scale contains only a few items (*n* = 6) to assess the CU traits and is limited with regard to the number of response options available (0 = not at all true, 1 = sometimes true, and 2 = definitely true), thus restricting the range of scores on the measure. Furthermore, many studies have indicated that the internal consistency of the CU factor is unacceptable [15, 16]. To overcome these limitations, the Inventory of Callous–Unemotional Traits (ICU) was introduced[17]. Each of the four items that loaded consistently on the CU factor of the APSD was expanded with six new items. Specifically, three positively (e.g., “Shows no remorse when he/she has done something wrong”) and three negatively worded (e.g., “Easily admits to being wrong”) items were developed from each original item, leading to a 24-item scale with equal number of items worded in each direction [18].

In addition, these items are developmentally appropriate for use with older children as well as adults (e.g., “I do not feel remorseful when I do something wrong” or “I do not like to put the time into doing things well”). Most recently, researchers have revealed that the ICU may be a promising measure that has some utility in adults [8, 11]. Still, validation of the structure of the ICU in various samples, specifically in non-Western cultures, is warranted. To this end, the aim of the present study was to examine the psychometric properties of the ICU in a Chinese sample of community adults.

### The factor structure of the ICU

Prior validation studies of the ICU with Western samples (adolescents and adults) have demonstrated differential factorial structures ranging from 2- to 5-factors [18–27]. In general, a three-factor bifactor model, in which all items load onto a general factor as well as onto three identified subfactors (i.e., Uncaring, Callousness, and Unemotional), has received most support in adolescents[20] [23, 25].

However, further examination of the bifactor model in those studies revealed that this model didn’t meet the common model fit criteria (i.e., CFI/TLI > .90 and RMSEA < .08, [28]). Table 1 summarizes findings of those studies that tested the factor structure of the ICU. It is worth noting that almost all studies accepted the bifactor model as the best fit model because it was better than the unidimensional model and the intercorrelated three-factor model (without a higher order general factor). However, detailed examination of the models indicated that the model fit was insufficient. For instance, Ciucci et al. (2014)[19] compared four different models of the self-report ICU in a sample of 540 Italian children. Although the bifactor model exhibited the best fit (*χ*^2^ = 442.06, *df* = 198, *χ*^2^/df = 2.23, CFI = .87, TLI = .85 and RMSEA = .05), none of the four models reached the minimum fit criteria [28]. Again using the self-report ICU, Feilhauer and colleagues (2012) [21] compared a one-factor model, a three-factor intercorrelated model, a three-factor hierarchical model, and a bifactor model in a mixed adolescent sample. All models failed to fit the data well; the authors therefore extracted five factors through exploratory factor analyses.

**Table 1:** Summary of study characteristics and best fit models reported in previous CFA studies of ICU.

Authors	Form	Sample characteristic (age-range)	Country	Method:CFA/EFA	Best model	Alpha (number of items)	Fit Indices
Benesch et al.(2014)	PR	131 boys with (ODD/CD); ages 6 to 12 years (*M* = 8.9; *SD* = 1.9).	Germany	CFA: ML, EFA	No model fit well; EFA yielded a new 3F model	Total .80(21) Callousness .81(11); Unconcern .73(5); Unemotional .76(5);	
Byrd et al. (2013)	SR	425 community adult males	USA	EFA, CFA: WLSMV	3F bifactor model with 5 correlated errors	Original scale: Total, .80(24); Callousness .70(11); Uncaring.84(8); Unemotional .55(5).	χ^2^ = 375.41, *df* = 78, CFI = .88, TLI = .91, RMSEA = .10
Colins et al.(2016)	SR	191 detained female adolescents (*M* age = 15.76, *SD* = 1.02).	Belgium	CFA: WLSMV	2F bifactor model without item 6	Total .76(11); Callousness .72(6); Uncaring .74(5)	χ^2^ = 58.51, *df* = 33, CFI = .96, TLI = .94, RMSEA = .06
Ciucci et al.(2014)	SR	540 community youths (52.6% girls); ages 10 to 16 years	Italy	CFA	3F hierarchical	Total .81(22); Callousness .66(9); Uncaring .72(8); Unemotional .64(5)	χ^2^ = 442.06, *df* = 198, CFI = .87, TLI = .85
Essau et al. (2006)	SR	1,443 adolescents (774 boys, 669 girls); ages 13 to 18 years	Germany	CFA: ML, EFA	Original 3F bifactor model	Total .77(24); Callousness .70(11); Uncaring .73(8); Unemotional .64(5)	χ^2^ = 1824.942, *df =* 228, GFI = .82, RMSEA = .10
Fanti et al.(2009)	SR	347 adolescents (49% girls); ages 12 to 18 years (M = 14.63)	Cyprus	CFA	3F bifactor model with 17 correlated errors	Total .81; Callousness .79; Unemotional .68; Uncaring .78	χ^2^ = 372.12, *df* = 212, SRMR = .05, CFI = .92, RMSEA = .05
Feilhauer et al.(2012)	SR	young clinical offenders (detained, *N* = 127), community (*N* = 172), non-clinical offenders (*N* = 42) and an externalizing non-offender group (*N* = 42); ages 13 to 20 years	Dutch	CFA: ML, EFA	No model fit well; EFA got 5 factors	Lack of Conscience .71(6); Lack of Empathy .48(5). Callousness .46 (5); Uncaring .72(4); Unemotional .63(4);	
Gao & Zhang (2016)	SR; PR	Community sample of 340 boys and girls; ages 8 to 10 years.	USA	CFA: ML	SR: modified 2F without Unemotional items, and1 correlated errors; PR: modified 3F with 8 correlated errors	SR: Total .7(13); Callousness .65(7); Uncaring .77(6). PR: Total .85(19); Callousness .71(7); Uncaring .83(8); Unemotional .63(4).	χ^2^ = 110.83, *df* = 63, CFI = .93, GFI = .95, RMSEA = .05; χ^2^ = 303.94, *df* = 141, CFI = .91, GFI = .92, RMSEA = .06
Hawes et al. (2014)	PR	250 boys exhibiting significant conduct problems; ages 6 to 12 years	USA	CFA: WLSMV, 3F-bifactor model failed to fit the data; IRT got 2F short form	2F 12 item short version	Total .85(12); Callousness .87(7); Uncaring .76(5).	3F bifactor: χ^2^ = 553.36, *df* = 228, CFI = .87, TLI = .84, RMSEA = .08; 2F 12 item short version:χ^2^ = 100.21, *df* = 53, CFI = .97, TLI = .96, RMSEA = .06; 2F bifactor: χ^2^ = 86.57, *df* = 43, CFI = .97, TLI = .96, RMSEA = .06.
Kimonis et al. (2008)	SR	248 juvenile offenders (188 boys, 60 girls); ages 12 to 20 years	USA	CFA: ML	3F bifactor model without items 2 and 10	Total .81(22); Callousness .80(9); Uncaring .81(8); Unemotional .53(5).	χ^2^ = 343.52, *df* = 187, CFI = .87, RMSEA = .06
Kimonis et al.(2013)	SR	687 college students (females 77.6%); ages 17 to 62 years (*M* = 21.3)	USA	PCA varimax rotation	New 3F	Total .81(22); Callousness .59(7); Uncaring .77(9); Unemotional .80(6).	
Kimonis et al.(2015)	PR	214 children (girls 48%); ages 3 to 6 years (*M* = 4.7)	Cyprus		Hawes et al. (2014) 2F model	Total .85(12); Callousness .82(7); Uncaring .80(5).	χ^2^ = 66.40, *df* = 53, CFI = .98, TLI = .98, RMSEA = .04
López-Romero et al.(2015)	SR	324 adolescents and young adults (72.5% males) from the Juvenile Justice System; ages 12 to 21 years (M = 16.13, *SD* = 1.98)	Spain	CFA: ULS	Revised 3F hierarchical model	Total .88(23); Callousness .76(10); Unemotional .78(5); Uncaring .82(8).	χ^2^ = 384.56, *df* = 229, GFI = .95, AGFI = .93, NFI = .91, RMR = .07
Moore et al. (2017)	PR	339 twin pairs (*N* = 678); ages 9 to 14 years.	USA	CFA: FIML	New 2F bifactor model		CFI = .986, TLI = .984, RMSEA = .044
Paiva-Salisbury et al.(2017)	SR	234 adolescents (191 juvenile offenders, 43 high school students; 63% male); ages 11 to 17 years	USA	CFA: robust ML	SF-ICU	Total .85(22); Callousness .74(9); Uncaring .80(8); Unemotional .70(5).	SF-ICU: χ^2^ = 105.66, *df* = 53,CFI = .92, RMSEA = .05
Pihet et al. (2015)	SR	397 community adolescents (38% males) *M* = 15.8 years (*SD* = 1.9); 164 institutionalized adolescents (70% males) *M* = 15.0 years (*SD* = 2.0)	Switzerland	CFA: ML	Original 3F bifactor model	For all samples: Total .79(24); Callousness .72(11); Uncaring .73(8); Unemotional .65(5).	χ^2^/*df* = 3.1, CFI = .83, RMSEA = .06
Roose et al. (2010)	SR TR PR	455 community adolescents (56% males); mean age = 16.67 years (*SD* = 1.34; range = 14.17–20.58)	Belgium	CFA: ML	Original 3F bifactor model		SR: χ^2^ = 674.53, *df* = 228, CFI = .92, AGFI = .86, GFI = .89, RMSEA = .07; PR: χ^2^ = 375.12, *df* = 228, CFI = .93, AGFI = .78, GFI = .83, RMSEA = .07; TR: χ^2^ = 534.03, *df* = 228, CFI = .90, AGFI = .64, GFI = .73, RMSEA = .11; Combined: χ^2^ = 348.31, *df* = 228, CFI = .96, AGFI = .74, GFI = .80, RMSEA = .07.
Waller et al.(2015)	PR	450 high-risk 9-year-olds	USA	CFA: WLSMV	Final 3F bifactor model without items 10 and 23. Items 8, 3, 5, and 13 were specified to have general variance but no specific variance and with 5 correlated errors	Total .87(22); Callousness .78(10); Uncaring .81(9); Unemotional .65(5)	χ^2^ = 603.32, *df* = 186, CFI = .95, RMSEA = .06; SF-ICU: χ^2^ = 126.98, *df* = 53, CFI = .98, RMSEA = .05
Willoughby et al. (2015)	PR	1,078 children (50% male); *M* age = 7.3, *SD* = 0.3 years	USA	CFA: WLSMV	New 2F model (EP, CU)		χ^2^ = 1447.7, *df* = 251, CFI = .94

SR = Self-Report; PR = Parent Report; TR = Teacher Report; ULS = Unweighted Least Squares; WLSMV = Robust Weighted Least-Squares with Mean and Variance Adjustment Estimator; ML = Maximum Likelihood; FIML = Full Information Maximum Likelihood; CFA = Confirmatory Factor Analysis; EFA = Exploratory Factor Analysis; CFI = Comparative Fit Index; TLI = Tucker-Lewis Index; RMSEA = Root Mean-Square Error or Approximation; GFI = Goodness of Fit Index

Only two studies have examined the factor structure of the original ICU in adults and the findings are inconsistent. Byrd et al. (2013) [8]in a community sample of adult males concluded that the three-factor bifactor model was the best, although the model fit indices did not reach the criteria (CFI = .88, TLI = .91, and RMSEA = .10). In a group of college students Kimonis et al. (2013) [11]conducted an exploratory principal components analysis with varimax rotation, and concluded that a new three-factor model was suitable with 37.6% of variance explained. In sum, the extant literature provides limited support to the bifactor model as representing the underlying dimensionality of the ICU, and much less is known in adults.

### The shortened forms of the ICU

Several studies have developed various shortened forms of the ICU after failing to achieve acceptable fit with original items. For example, Hawes and colleagues (2014a) [29]examined the factor structure of the ICU in 250 boys who exhibited significant conduct problems. With the three-factor bifactor model failing to fit their data, a 12-item shortened form was developed through item response theory. This shortened form of the ICU (SF-ICU) consists of two factors: callousness (7 items) and uncaring (5 items), and its scores demonstrated good reliability and discrimination across the continuum of the CU constructs [29]. The total score of the SF-ICU exhibited the expected associations with relevant external measures, including conduct problems (*r* = .46, *p* < .01) and social competence (*r* = -.55, *p* < .01).

Since then, several independent research groups successfully replicated its factor structure in various youth samples [30–33]. For example, in a sample of detained female adolescents, Colins et al. found that the SF-ICU fit the data well. Similarly, Waller et al. (2015) [33] examined the factor structure of the ICU parent version in 540 high-risk 9-year-olds adolescents and reported that a modified three-factor bifactor model fit well (χ^2^ = 603.32, *df* = 186, CFI = .95, RMSEA = .06). Noticeably, in that study the SF-ICU fit the data equally well (χ^2^ = 126.98, *df* = 53, CFI = .98, RMSEA = .05). Similar findings were reported by Paiva-Salisbury et al. (SF-ICU: χ^2^ = 105.66, *df* = 53, CFI = .92, RMSEA = .05). Moreover, those studies extended the construct validity of the SF-ICU through examining theoretically relevant variables, including rule-breaking behavior, aggression, and attention problems.

More recently, a new shortened version of the ICU composed of ten items (ICU-10; i.e., item 3, 5, 7, 8, 11, 15, 16, 17, 23 and 24) has been proposed as a one-dimension scale to assess the overall CU traits through item response theory analyses in adolescents with conduct problems [34]. In this model, seven of the ten items (3, 5, 15, 16, 17, 23 and 24) comprise the uncaring subscale, and the remaining three items (7, 8, and 11) comprise the callousness factor. The ICU-10 had good *α* coefficient (α = .78) and test-retest reliability over 6 months (*r* = .59). Regarding the criterion validity, the ICU-10 total score was significantly associated with empathy (*r* = -.40, *p* < .01), delinquency (*r* = .30, *p* < .01), school misconduct (*r* = .32, *p* < .01), and proactive (*r* = .25, *p* < .01) and reactive aggression (*r* = .27, *p* < .01) [34]. More importantly, those correlations with the ICU-10 were similar to the correlations with the original ICU (excluding items 2 and 24). Furthermore, six items (i.e., 5, 8, 11, 16, 17, and 24) of the SF-ICU overlapped with the ICU-10.

In a sample of male and female children from the community, Gao and Zhang (2016) [35]created two different shortened versions for the child- and parent-report forms of the ICU. Specifically, the child self-report shortened form (ICU-13) consists of 13 items that were divided into two factors: callousness (7 items) and uncaring (6 items). The *α* coefficients for the ICU-13 total score and the two subfactor scores were acceptable. In addition, the ICU-13 total score and its two subfactor scores exhibited the expected associations with relevant external measures [35]. In sum, shortened forms of the ICU have recently received promising initial support. However, none has examined the validity of shortened forms in adults or in non-Western samples.

### The current study

The primary aim of the current study was to examine the factor structure of the original 24-item ICU and the shortened forms of the ICU (i.e., SF-ICU, ICU-10, and ICU-13) in Chinese adults from the community. To this end, a series of confirmative factor analyses (CFA) were conducted to compare these models. The model specifications are present in Table 2.

**Table 2:** Model specification for tested models.

Model Number	Model Specification and Items	Cronbach’s*α*	MIC	Number of Items	Author
M1 M2 M3	callousness: 2 4 7 8 9 10 11 12 18 20 21	.75	.21	11	Essau et al. (2006)
	uncaring: 3 5 13 15 16 17 23 24	.68	.21	8	
	unemotional: 1 6 14 19 22	.66	.28	5	
M4	callousness: 7 9 11 12 18 20	.67	.25	6	Gao & Zhang (2016)
	uncaring: 3 13 15 16 17 23 24	.67	.23	7	
	unemotional: 1 14 19 22	.58	.26	4	
M5	callousness: 2 4 7 8 9 11 12 18 20 21	.75	.23	10	López-Romero et al. (2013)
	uncaring: 3 5 13 15 16 17 23 24	68	.21	8	
	unemotional: 1 6 14 19 24	.66	.28	5	
M6	callousness: 2 4 7 9 11 12 18 20 21	.73	.24	9	Waller et al. (2015)
	uncaring: 15 16 17 24	.58	.26	4	
	unemotional: 1 6 14 19 22	.66	.28	5	
M7 M8	callousness: 4 5 8 9 12 13 16 17 18 21 24	.75	.22	11	Benesch et al. (2014)
	uncaring: 3 11 15 20 23	.70	.32	5	
	unemotional: 1 6 14 19 22	.66	.28	5	
M9	callousness: 2 4 7 9 11 18 20 21	.70	.23	8	Kimonis et al. (2013)
	uncaring: 3 5 8 13 15 16 17 23 24	.70	.21	9	
	unemotional: 1 6 10 14 19 22	.64	.22	6	
M10 M11	EP: 2 4 6 7 9 11 12 18 20 21 22	.72	.19	11	Willoughby et al. (2015)
	CU: 1 3 5 8 10 13 14 15 16 17 19 23 24	.69	.15	13	
M12	Unemotional: 1 6 14 19 22	.66	.28	5	Moore et al. (2017)
	Callous / Uncaring: 3 7 11 15 20 23	.68	.26	6	
M13 M14	callousness: 4 6 9 11 12 18 21	.66	.20	7	Hawes et al. (2014) SF-ICU
	uncaring: 5 8 16 17 24	.59	.22	5	
M15	callousness: 4 9 11 12 18 21	.69	.27	6	Colins et al. (2016); ICU-11
	uncaring: 5 8 16 17 24	.59	.22	5	
M16	callousness: 4 7 8 9 11 12 18 21	.74	.26	8	Houghton et al. (2013)
	uncaring: 3 5 13 15 16 17 23 24	.68	.21	8	
M17	callousness: 7 9 11 12 18 20 21	.71	.26	7	Gao & Zhang (2016)
	uncaring: 3 13 15 16 17 24	.62	.22	6	
M18	ICU: 3 5 7 8 11 15 16 17 23 24	.70	.19	10	Ray et al. (2016); ICU-10

EP = Empathic-Prosocial; CU = Callousness-Unemotional; ICU = the Inventory of Callous–Unemotional Traits; MIC = Mean inter-Item Correlations. The M3, M8, M11, M14 are bifactor version of models corresponding to M2, M7, M10 and M13, respectively. M12: all 24 items loading on the general factor, meanwhile 5 and 6 items loading on Unemotional and Callous/Uncaring factor respectively, general factor and two specific factors uncorrected with each other.

On the basis of findings from recent studies, we predicted that the bifactor model of the original ICU would provide unacceptable fit to the data. We also would explore which of the shortened version fit our data best, given that no such study has been done in adult populations. Additionally, we aimed to test the construct validity of the best-fitted model by examining whether the total and factor scores were correlated as expected with constructs including (a) alternative measures of psychopathy (i.e., the Levenson Self-Report Psychopathy Scale (LSRP)); (b) aggression (e.g., Reactive–Proactive Aggression Questionnaire); (c) antisocial personality symptoms, and (d) trait measures of empathy and callousness. Based on previous research, we expected that CU traits would be positively related to the LSRP total and subscale scores, in particular the primary psychopathy subscale score [11], reactive and proactive aggression scores [20] [21, 23], and the number of antisocial personality symptoms [11]. In addition, we expected that the CU traits would be positively related to the scores on trait measures of callousness, and negatively correlated with empathy [11]. Additionally, the ICU-callousness factor score would be preferentially associated with the trait measures of callousness.

## Materials and methods

### Participants

Two independent samples of participants were recruited from a community college in Guangzhou City, China. The first sample consisted of 345 participants (62.9% female, *n* = 217), who ranged in age from 19 to 52 years old (*M* = 25.98, *SD* = 5.79). The second sample consisted of 292 participants (63% female, *n* = 184), who ranged in age from 18 to 48 years old (*M* = 27.04, *SD* = 5.16). With regard to racial distribution, 99.0% of the participants were Han, the largest race in China. Different measures were administered to each sample (see below).

Questionnaires were administered to only those who had given informed consent. This study was approved by the Human Subjects Review Committee at Guangzhou University. Participants completed surveys in school during specific class periods lasting approximately 40 minutes. After answering basic demographic questions (e.g., age, sex, race/ethnicity), participants completed the measures described below. All questionnaires were administered in Chinese.

### Measures—Both samples

#### Inventory of Callous-unemotional traits

The Chinese version of the ICU[17] was translated into Chinese and back-translated to English to ensure accuracy. The translators further discussed items with translation differences, until they reached an agreement. The questionnaires were then piloted in a different sample (*n* = 22) of college students to assess for readability, and no further revision was needed.

#### Personality diagnostic questionnaire

The Antisocial Personality Disorder (ASPD) subscale of the PDQ-4 [36, 37]was used to measure characteristics of ASPD in both samples. The ASPD scale consists of 22 forced-choice items that are rated as either true or false. Items correspond to diagnostic criteria for the ASPD from the Diagnostic and Statistical Manual of Mental Disorders[38] (4th ed., American Psychiatric Association, 2000). Sample items include “I’ve been in trouble with the law several times,” and “Lying comes easily to me and I often do it.” The Chinese version of the PDQ-4 has demonstrated moderate internal consistency (α coefficients ranged from .56 to .78) and test- retest reliability (the coefficients ranged from .49 to .80) in college students [37]. In the current sample, the internal consistency was .66.

#### IPIP-empathy and IPIP-Callousness

An established 10-item Likert-scale questionnaire assessing empathy was drawn from the International Personality Item Pool (IPIP). Sample items include “Suffer from others' sorrows” and “Don't understand people who get emotional”. Scales drawn from the IPIP have well-established reliability and validity in the literature, and are freely available (http://ipip.ori.org/). Ten items correspond to the empathy subscale of Jackson Personality Inventory-Revised [39], and the coefficient α was .80 in initial sample. The Chinese version of the IPIP-Empathy was translated in the current study and the coefficient α was .73.

Similarly, an established 7-item Likert-scale questionnaire assessing callousness was drawn from the IPIP. Sample items include “Am not a caring person” and “Can't be bothered with others’ needs”. The IPIP-Callousness scale has been scrutinized in community (N = 1,269) and patient samples (N = 628), and the coefficient αs were .85 and .83, respectively [40]. The Chinese version of the IPIP-Callousness was translated in the current study, and the internal consistency was .78.

### Measures—Sample one only

#### The Reactive–Proactive Aggression Questionnaire (RPQ)

The RPQ[41] is a 23-item self-report questionnaire that distinguishes between proactive and reactive aggression. A total of 12 items assess proactive aggression (e.g., “Hurt others to win a game”), and 11 items assess reactive aggression (e.g., “Reacted angrily when provoked by others”). Items are scored on a three-point scale (0 = never, 1 = sometimes, 2 = often), and scores of relevant items are summated to form measures of reactive or proactive aggression together with an overall score of total aggression. The questionnaire has high internal consistency and good validity [41]. Prior studies in Chinese samples have shown excellent internal consistency and good factorial validity and construct validity [42, 43]. In the current study, consistency measures were comparable, with a coefficient α of .90 for the total scale, .80 for the reactive, and .86 for the proactive aggression subscale, respectively.

### Measures—Sample two only

#### The Levenson Self-Report Psychopathy Scale (LSRP)

The LSRP[44] is a 26-item self-report questionnaire that provides a total score of psychopathy and subscale scores for primary and secondary psychopathy, respectively. The Likert-style items have four response options ranging from 1 (strongly disagree) to 4 (strongly agree). Research has indicated that this measure has adequate reliability, with coefficient αs ranging from .63 to .82 for the two subscales [44]. The Chinese version of the LSRP was created and validated in a sample of Chinese inmates [45]. In that study, the original two-factor structure fit the data reasonably well and provided good construct validity. In the current study, the coefficient αs for the total and factor scores were .78, .68 and .76, respectively.

#### The aggression questionnaire

The AQ [46]is a 29-item questionnaire assessing aggression in three components: a behavioral component represented by the subscales of physical aggression and verbal aggression, an emotional component covered by the anger subscale, and a cognitive component represented by hostility. Items were scored on a 5-point Likert scale from 1 (extremely unlike me) to 5 (extremely like me). The Chinese revision of the AQ has good internal consistency ranging from .60 to .89 and appropriate construct validity[47]. In the present study, the internal consistency was acceptable to good for the four subscales and the total scale, ranging from .60 to .89.

### Statistical analyses

To compare the various models of the ICU, a series of CFAs were conducted via Mplus 7.0[48] using robust weighted least-squares with a mean and variance adjustment (WLSMV) estimator. This method is strongly recommended for data with ordinal items [48]. Following generally accepted practice, we evaluated the fit of each model by examining multiple fit indices [28], including Chi-square, the root-mean-square error of approximation (RMSEA), the Tucker-Lewis index (TLI), and the comparative fit index (CFI). Conventional guidelines suggest that RMSEA values ≤ .08 indicate acceptable model fit and ≤ .05 indicate good model fit, and CFI, TLI ≥ .90 indicate adequate model fit [28].

To evaluate the internal consistency of the ICU scores, Cronbach’s αs were calculated and coefficients were evaluated as follows: < .60 = insufficient; .60 to .69 = marginal; .70 to .79 = acceptable; .80 to .89 = good; and .90 or higher = excellent [49]. Given that α depends on inter-item correlations and number of items, we also calculated mean inter-item correlations (MIC), which is considered to be a more straightforward indicator of a scale’s internal consistency than Cronbach’s α and should be at minimum in the range of .15 to .50 to be considered adequate [50].

Finally, zero-order correlations were examined between ICU subscale scores and criterion variables (i.e., LSRP, RPQ, ASPD, AQ, and IPIP empathy and callousness). Additionally, to further evaluate the distinctive/independent contributions of the subscale scores of the ICU, we performed separate regression analyses, using subscale scores as predictors for each criterion variable.

## Results

### Confirmatory factor analysis

Table 3 summarizes the fit indices of these competing models in the whole sample. The original 3-factor model (M1) fit the data inadequately (WLSMV; χ2 = 1149.93, *df* = 249, *p* < .001, CFI = .83, TLI = .81, RMSEA = .08). After deleting item 2 and item 10 that were poorly correlated with the total score in previous studies [23] and also in the current sample, the revised model (M2) still showed poor fit. Two modified three-factor bifactor models displayed adequate fit according to the CFI (>.9) and RMSEA (< .08). The first model (M6) was proposed by Waller and colleagues based on the parent report version (WLSMV; χ2 = 592.27, *df* = 186, *p* < .001, CFI = .91, TLI = .89, RMSEA = .06), and the other model (M8) was reported by Benesch and colleagues (WLSMV; χ2 = 563.73, *df* = 168, *p* < .001, CFI = .92, TLI = .89, RMSEA = .06). Unfortunately, none of the other models that were based on the original 24 items fit the data well. For example, the new modified 2F bifactor model proposed by Moore et al. (2017) [51] exhibited unacceptable fit (χ2 = 1044.53, *df* = 241, *p* < .001, CFI = .84, TLI = .82, RMSEA = .07).

**Table 3:** Goodness-of-fit indices for tested models in the confirmatory factor analyses.

Model	N. of items	χ^2^	*df*	CFI	TLI	RMSEA [90%CI]	Author
*Full-scale models*							
(M1) Original 3F	24	1149.93	249	.83	.81	.08 [.07, .08]	Essau et al. (2006)
(M2) Original 3F without items 2 & 10	22	901.38	206	.86	.84	.07 [.07, .08]	
(M3) Original 3F bifactor without items 2 & 10	22	716.19	187	.89	.87	.07 [.06, .07]	
(M4) modified 3F PR	19	693.77	149	.86	.84	.08 [.07, .08]	Gao & Zhang (2016)
(M5) López-Romero modified 3F hierarchical	23	939.64	227	.86	.84	.07 [.07, .08]	López-Romero et al. (2013)
(M6) Waller-modified 3F bifactor	22	592.27	186	.91	.89	.06 [.05, .06]	Waller et al. (2015)
(M7) Benesch-modified 3F	21	708.22	186	.89	.87	.07 [.06, .07]	Benesch (2014)
(M8) Benesch-modified 3F bifactor	21	563.73	168	.92	.89	.06 [.06, .07]	Benesch et al. (2014)
(M9) Kimonis EFA 3F	23	1251.60	227	.79	.76	.08 [.08, .09]	Kimonis et al. (2013)
(M10) Willoughby 2F	24	1859.04	251	.69	.66	.10 [.10, .11]	Willoughby et al. (2015)
(M11) Willoughby 2F bifactor	24	1422.91	228	.77	.72	.09 [.09, .10]	
(M12) Moore-new 2F bifactor	24/11	1044.53	241	.84	.82	.07 [.07, .08]	Moore et al. (2017)
*Shortened-scale models*							
**(M13) SF-ICU**	**12**	**138.75**	**53**	**.96**	**.95**	**.05 [.04 .06]**	Hawes et al. (2012)
**(M14) SF-ICU bifactor**	**12**	**106.44**	**43**	**.97**	**.96**	**.04 [.04, .06]**	
**(M15) ICU-11**	**11**	**108.18**	**43**	**.97**	**.96**	**.05 [.04, .06]**	Colins et al. (2016)
(M16) Houghton-modified 2F	16	528.49	103	.89	.87	.08 [.07, .09]	Houghton et al. (2013)
(M17) Gao-modified 2F	13	436.24	64	.88	.85	.10 [.09, .10]	Gao & Zhang (2016)
(M18) ICU-10	10	286.62	35	.86	.82	.11 [.10, .12]	Ray et al. (2016)

*Note*: χ2 = chi-square; *df* = Degree of Freedom; CFI = comparative fit index; TLI = Tucker-Lewis index; RMSEA = Root Mean-Square Error of Approximation. Best-fitting models were shown in bold font.

For the shortened versions, the two-factor model (M13, with 12 items) created by Hawes et al. (2014a)[29] and its bifactor form (M14) displayed better fit compared to the other shortened models, and all items had statistically significant and moderate- to large-sized factor loadings on their respective factors (λ = .48-.69, *ps* < .01), with the exception of item 6 (λ = .14, *p* < .01). Similar findings have been reported by others [30].

After deleting item 6, the modified model (M15) fit the data very similar to M14 (see Table 4). Finally, the other short form models (M16-M18) fit the data inadequately. Thus, the modified model (M15) without item 6 was considered the best-fitting model and used in following analyses. To compare the differences in correlations between original ICU and this best-fitting model (ICU-11) with relevant variables, the original 3-factor model of ICU was used.

**Table 4:** Descriptive statistics and correlations among ICU factors and external criteria in the current sample.

	M	SD	Range	Skewness	Kurtosis	Cronbach’s *α*
ICU (24 items) Total	19.66	7.41	1–45	.34	.03	.80
uncaring	5.21	3.09	0–16	.45	-.27	.68
callousness	7.38	4.26	0–24	.59	.24	.75
unemotional	7.04	2.63	0–15	-.06	.18	.66
ICU-11	5.78	4.09	0–21	.66	-.01	.76
uncaring	2.56	2.07	0–10	.65	-.34	.59
callousness	3.23	2.64	0–13	.82	.51	.69
ASPD ^a^	2.25	2.22	0–11	1.07	.72	.66
LSRP ^b^	53.12	8.23	32–79	.13	.04	.78
LSRP-Primary ^b^	20.60	4.03	10–32	.18	.12	.68
LSRP-Secondary ^b^	32.49	6.13	19–53	.14	-.33	.76
Callousness-IPIP ^c^	13.31	4.04	7–27	.58	.20	.78
Empathy-IPIP ^c^	36.86	5.13	17–50	-.28	.28	.73
RPQ-P ^a^	1.61	2.31	0–22	3.37	20.16	.86
RPQ-R ^a^	6.41	3.25	0–18	.43	.44	.80
AQ-Verbal ^b^	10.82	2.31	5–25	.77	.48	.68
AQ-Physical ^b^	18.64	5.13	9–43	1.0	.74	.83
AQ-Anger ^b^	13.30	4.94	7–35	1.16	1.03	.85
AQ-Hostility ^b^	14.72	3.88	8–40	1.38	2.72	.75
AQ Total ^b^	57.15	12.50	29–105	1.08	.96	.89

*Note*: ASPD = The Antisocial Personality Disorder (ASPD) scale of the PDQ-4; LSRP Primary = Levenson Self-Report Primary Psychopathy Scale; LSRP-Secondary = Levenson Self-Report Secondary Psychopathy Scale; RPQ = the Reactive–Proactive Aggression Questionnaire; RPQ-P = the Proactive subscale of the RPQ; RPQ-R = the Reactive subscale of RPQ; AQ = the Aggression Questionnaire; AQ-Physical = the Physical aggression of AQ; AQ-verbal = the verbal aggression of AQ; AQ-Anger = the Anger subscale of AQ; AQ-Hostility = the Hostility subscale of AQ. Empathy-IPIP = Empathy scale selected from the International Personality Items Pool; Callousness-IPIP = Callousness scale selected from the International Personality Items Pool; ICU = Inventory of Callous-Unemotional Traits; SF-ICU = Shorten Form of ICU.

*a =* correlations for sample 1

*b* = correlations for sample 2

*c* = correlations for whole sample.

### Internal consistency and intercorrelations

The coefficient αs for all tested models in the current study are present in Table 2. Overall, the coefficient αs for the callousness factor were acceptable (αs >.70), and higher than those for the other two factors. Specifically, the coefficient α for the ICU total score (24 items) was .80 (MIC = .15). The reliability of the original model was .75 (MIC = .21), .68 (MIC = .21), and .66 (MIC = .28) for the callousness, uncaring, and unemotional subscale, respectively. The uncaring factor showed the strongest correlation with the callousness factor (*r* = .53), followed by the unemotional factor (*r* = .21). The callousness and unemotional factor showed the weakest correlation (*r* = .12). Inter-factor correlations after correcting for unreliability in CFA models were .75 for callousness-uncaring, .20 for callousness-unemotional, and .29 for uncaring-unemotional. Those correlations suggested that callousness strongly relates to uncaring, whereas the correlations between unemotional and other factors are moderate at most (< .30).

The coefficient αs for the ICU-11 total score, callousness, and uncaring were .76 (MIC = .22), .69 (MIC = .27) and .59 (MIC = .22), respectively. The correlation between the two subfactors was .51 at observed variable level; in contrast, the correlation reached .79 at latent variable level.

### Construct validity

Descriptive statistics and internal consistency estimates of all measures in the current sample are presented in Table 4.

The zero-order correlations were calculated to examine the associations between ICU-11 total and subfactor scores and external criteria measures (see Table 5). As expected, significant correlations were found between ICU-11 total and LSRP total scores (*r* = .50, *p* < .01), as well as IPIP-callousness and empathy scores (*r* = .63 and *r* = –.40, *p*s < .01, respectively). The ICU-11 scores showed much stronger correlations with the LSRP primary than with the secondary psychopathy scores. In addition, ICU-11 scores showed significant correlations with measures of antisocial personality symptoms (i.e., ASPD). The correlations between ICU-11 scores and aggression measures were moderately significant, with the strongest associations emerging between proactive aggression and the callousness factor. Finally, ICU-11 scores were not significantly associated with verbal or physical aggression, although their associations with anger and hostility were significant.

**Table 5:** Correlational and regression analyses between ICU and external criteria measures.

	Short form ICU (11 items)			Original ICU (24 items)			
	Uncaring	Callousness	ICU Total	Uncaring	Callousness	Unemotional	ICU Total
ASPD ^a^	.17**(-.01)	.30**(.26**)	.28**	.17**(.01)	.32**(.32**)	.03(-.01)	.27**
LSRP Total ^b^	.41**(.23**)	.44**(.34**)	.50**	.50**(.32**)	.50**(.34**)	.04(-.043)	.52**
LSRP-Primary ^b^	.46**(.27**)	.48**(.36**)	.55**	.46**(.27**)	.49**(.36**)	.08(.00)	.51**
LSRP-Secondary ^b^	.14*(.07)	.16**(.137*)	.18**	.31**(.23**)	.27**(.17*)	-.04(-.08)	.29**
IPIP-Callousness ^c^	.48**(.24**)	.60**(.48**)	.63**	.48**(.19**)	.60**(.49**)	.23**(.15**)	.64**
IPIP-Empathy ^c^	-.36**(-.25**)	-.33**(-.21**)	-.40**	-.38**(-.22**)	-.32**(-.18**)	-.36**(-.32**)	-.49**
RPQ-P ^a^	.24**(.12*)	.30**(.22**)	.30**	.20**(.08)	.29**(.26**)	-.02(-.08)	.24**
RPQ-R ^a^	.05(-.05)	.17**(.18**)	.13*	.08(.001)	.21**(.22**)	-.10(-.13*)	.11*
AQ-Verbal ^b^	-.01(-.10)	.10(.18*)	.08	.01(-.06)	.143*(.(19**)	-.22**(-.22**)	.01
AQ-Physical ^b^	.01(-.06)	.13*(.14)	.07	.04(-.05)	.13*(.17*)	-.09(-.09)	.07
AQ-Anger ^b^	.14*(.03)	.23**(.22**)	.22**	.20*(.05)	.14*(35**)	-.28**(-.30**)	.19**
AQ-Hostility ^b^	.17**(.03)	.28**(.27**)	.27**	.29**(.12)	.36**(.32**)	-.04(-.05)	.34**
AQ Total ^b^	.11(-.03)	.24**(.25**)	.21**	.18*(.02)	.32**(.34**)	-.19**(-.21**)	.21**

*Note*: Standardized Beta Coefficients are given in parentheses. ASPD = the Antisocial Personality Disorder (ASPD) scale of the PDQ-4; LSRP Primary = Levenson Self-Report Scale, primary psychopathy subscale; LSRP-Secondary = Levenson Self-Report Scale, secondary psychopathy subscale; RPQ = the Reactive-Proactive Aggression Questionnaire; RPQ-P = the Proactive subscale of the RPQ; RPQ-R = the Reactive subscale of the RPQ; AQ = the Aggression Questionnaire; AQ-Physical = the Physical aggression subscale of the AQ; AQ-verbal = the verbal aggression subscale of the AQ; AQ-Anger = the Anger subscale of the AQ; AQ-Hostility = the Hostility subscale of the AQ. IPIP-Empathy = the Empathy scale from the International Personality Items Pool; IPIP-Callousness = the Callousness scale from the International Personality Items Pool; ICU = Inventory of Callous-Unemotional Traits.

*a =* correlations for sample 1

*b* = correlations for sample 2

*c* = correlations for the whole sample.

*p< .05 (2-tailed).

**p< .01 (2-tailed).

After entering both ICU-11 factor scores in the regression, only callousness was significantly related to ASPD (*β* = .26, *p* < .01), LSRP secondary psychopathy (*β* = .14, *p* < .01), reactive aggression (*β* = .18, *p* < .01), anger (*β* = .22, *p* < .01), and hostility (*β* = .27, *p* < .01).

Correlations between the original ICU (24 items) total and factor scores, and external variables were largely consistent with those for the ICU-11 (see Table 5). It is worth noting that the unemotional factor showed weaker or no associations with the external variables, except that it demonstrated stronger associations with scores on IPIP-empathy, verbal aggression, and anger.

## Discussion

The developmental progression of the CU traits from childhood and adolescence to adulthood has received increasing attention of late. Therefore, an efficient, reliable, and valid measure of the CU traits covering various ages is of the upmost priority [52]. Although evidence from previous validation studies has shown that the ICU may be a promising measure in adult populations [18, 20, 21, 23, 26], these studies were restricted to the samples and findings on the factorial structure of the ICU have been inconsistent. To the authors’ knowledge, the current study is the first to compare various models of the ICU and their psychometric properties in a non-Western adult sample. In particular, this is the first study to compare five recently proposed shortened forms of ICU. In general, we found limited evidence to support the original three-factor bifactor model in our sample. Instead, a shortened version with 11 items (e.g., ICU-11) loaded onto two factors (i.e., callousness and uncaring) demonstrated good fit and reasonable construct validity. Finally, our findings also revealed that the ICU-11 exhibits similar correlations with external variables as compared with the original ICU, suggesting that it this shortened form could be used as a reliable and valid measure of CU traits in community adults.

Consistent with most previous studies [8], the bifactor structure fit our data better as compared with the single-factor and the three correlated factor models, although its fit indices were still unacceptable. Other three-factor models with various modifications [33] were also tested but none provided good model fit. Taken together, we may conclude that at least at the item level, limited evidence supports the three-factor bifactor model in non-Western adult populations.

The coefficient *α* for the unemotional subscale was only .66 in the current sample. Similar issue with the unemotional factor has been reported in Byrd et al. (2013), Essau et al. (2006), and Kimonis et al. (2008) (coefficient *α* = .55, .64 and .53, receptively)[8, 18, 23]. In addition, the unemotional factor of the original ICU showed a weaker or negligible association with the majority of the variables except for IPIP-empathy, AQ-verbal aggression, and AQ-anger. Of note, Feilhauer et al. (2012) [21] also failed to find significant associations between the unemotional factor and scores on the ASPD and the PCL: YV. Furthermore, in our sample we found null or negative associations between the unemotional factor and aggression measures, a finding seemed unexpected at the first glance. For example, Feilhauer et al. (2012)[21] found that the unemotional factor was positively associated with aggression as assessed by the AQ and RPQ, in a mixed sample aged from 13 to 20 years. Indeed, the correlation coefficients ranged from .18 to .30 (*p*s < .01). However, a more careful examination of the constituent items of the anger factor revealed substantial overlap with the items in the unemotional factor. For example, the two items of the anger factor, namely “I have trouble controlling my temper” and “Sometimes I fly off the handle for no good reason”, capture the expression of anger emotion; meanwhile, the items in the unemotional factor, namely “I express my feelings openly (reversed)” and “I do not show my emotions to others”, reflect the concealment of emotion. Therefore, it is not surprising that the anger factor of the Aggression Questionnaire was found to be negatively associated with the unemotional factor score (*r* = –.29, *p* < .001). Taken together, more research on the unemotional factor is warranted.

Given that the unemotional factor displayed poor reliability and unexpected associations with theoretically related variables [22, 29, 30], some authors eliminated the unemotional items to develop a shortened form of the ICU. In general, the shortened version proposed by Hawes et al. (2014a) [29]consists of 12 items loaded onto two factors (i.e., callousness and uncaring) and fit our data well. Notably, in line with recent work [30], item 6 (i.e., “Does not show emotions”) demonstrated poor factor loading (λ = .14) and was subsequently deleted from the analyses. In fact, item 6 was not included in any of the two-factor models (except [29]).

With regard to internal consistency, the findings of the current study were consistent with most previous studies [8, 18, 23]. Specifically, the coefficient *α*s for the callousness subscale in most of the tested models were acceptable, whereas the uncaring factor demonstrated poor internal consistency. Notably, the coefficient *α* for the uncaring factor in Hawes et al.’ model was only .59, which was much lower than findings in other reports [29, 30], although the MICs were in a reasonable range (>.15). Such low internal consistency indicates that the items in the uncaring factor need to be further refined in future studies.

The ICU-11 total score exhibited robust associations with other measures of psychopathic features. Specifically, the primary psychopathy factor of the LSRP assesses a callous, manipulative, and self-centered lifestyle, while the secondary psychopathy factor assesses impulsivity and poor behavior controls [44]. As expected, the ICU-11 total score showed stronger correlations with the primary than with the secondary psychopathy scores, which is in line with previous studies in adults[11]. Furthermore, the ICU-11 total score showed stronger correlations with psychopathy scores (i.e., LSRP) than with the number of antisocial personality symptoms (i.e., ASPD; *r* = .52 vs. *r* = .27), indicating that these characteristics are related to but distinct from symptoms associated with antisocial personality disorder. Additionally, in line with prior studies[30, 31, 33], the correlation pattern for the total score of the ICU-11 highly agrees with that for the original ICU, suggesting that this shortened scale keeps sufficient information from its original form.

At the factor level, both callousness and uncaring factor scores correlated significantly with overall scores on the ASPD, LSRP, and proactive aggression, again in line with previous findings [11, 26]. Interestingly, when both factors were entered in regression, only callousness was significantly related to these measures. Moreover, the construct validity of the ICU-11 was supported by its associations with measures of empathy and callousness. Again, the pattern of correlations among ICU-11 callousness/uncaring and external measures was similar to that with the original ICU.

The current study has several strengths, including it being the first to compare an extensive list of models proposed across various studies. In addition, it utilized a large sample of Chinese adults to assess the psychometric properties of the ICU, and was the first to examine five recently developed shortened versions. There are, however, several limitations to this study. First, this study relied on self-reports for both the measure of CU traits and the scales used to assess its validity. Therefore, the correlations could be inflated by shared method variance. Moreover, assessing psychopathic traits through self-reports has long been viewed with skepticism [53, 54], although recent studies have shown that self-report assessments of psychopathic-like traits are reliable and valid [55, 56]. Future investigations would benefit from using a multi-informant approach, including assessments from partners and/or friends. Second, there were several external correlated instruments that demonstrated less than ideal internal consistency (i.e., Cronbach’s *α* below .80), therefore, those findings need to be further scrutinized in future investigations.

Overall, our results indicated that the original three-factor model and its variant (correlated three factor, three-factor bifactor, and three factor hierarchical) displayed poor overall fit in a group of Chinese community adults. A newly proposed 11-item model consisting the callousness and uncaring factors had excellent overall fit. The internal consistency was good to acceptable for the total and the two factors scores. Correlations with a wide range of external variables provided preliminary evidence to support the construct validity of the ICU-11. Altogether, our findings support the cross-cultural generalizability of the shortened two-factor model of the ICU in non-Western and non-institutional adult populations.

## Supporting information

S1 Appendix The inventory of callous-unemotional traits Chinese version. (DOCX)

S1 Table Datasets sample 1. (XLSX)

S2 Table Datasets sample 2. (XLSX)
