# Supplementary material for: The factor structure and construct validity of the inventory of callous-unemotional traits in Chinese undergraduate students
Source: PLoS One. 2017 Dec 7;12(12):e0189003. doi: 10.1371/journal.pone.0189003 (PMC5720694; doi:10.1371/journal.pone.0189003)
Supplement: S1 Appendix — (DOCX) [file pone.0189003.s001.docx]

ICU

**指导语：下面是一些关于你个性特点的描述，请根据与你实际情况描述相符的程度，在题目后面的数字上画圈“○”，其中“1=完全不符合”，“2=有点符合”，“3=符合”，“4=完全符合”**

| **序号** | **内容** | **完全**  **不符合** | **有点**  **符合** | **符合** | **完全**  **符合** | |
| --- | --- | --- | --- | --- | --- | --- |
| 1 | 我会将自己的情绪表现出来。 | 1 | 2 | 3 | 4 | |
| 2 | 我认为对的事，别人却认为是错误的。 | 1 | 2 | 3 | 4 | |
| 3 | 我在意自己在学校或工作上的表现。 | 1 | 2 | 3 | 4 | |
| 4 | 为了达到目的，我不在乎伤害了谁。 | 1 | 2 | 3 | 4 | |
| 5 | 犯了错，我会感到难过或内疚。 | 1 | 2 | 3 | 4 | |
| 6 | 我不会向别人坦露内心情绪和感受。 | 1 | 2 | 3 | 4 | |
| 7 | 我不担心会不会迟到。 | 1 | 2 | 3 | 4 | |
| 8 | 我在意别人的感受。 | 1 | 2 | 3 | 4 | |
| 9 | 我不在意是否惹了麻烦。 | 1 | 2 | 3 | 4 | |
| 10 | 我能控制自己的情绪。 | 1 | 2 | 3 | 4 | |
| 11 | 我不在意事情是否做得足够好。 | 1 | 2 | 3 | 4 | |
| 12 | 我似乎很冷淡也不关心别人。 | 1 | 2 | 3 | 4 | |
| 13 | 我乐于承认错误。 | 1 | 2 | 3 | 4 | |
| 14 | 别人很容易觉察到我的情绪变化。 | 1 | 2 | 3 | 4 | |
| 15 | 我总是尽力而为。 | 1 | 2 | 3 | 4 | |
| 16 | 我会向被我伤害的人道歉（说“对不起”）。 | 1 | 2 | 3 | 4 | |
| 17 | 我尽力不去伤害别人的感情。 | 1 | 2 | 3 | 4 | |
| 18 | 做错了事，我不会感到懊悔。 | 1 | 2 | 3 | 4 | |
| 19 | 我表情丰富而且容易情绪化。 | 1 | 2 | 3 | 4 | |
| 20 | 我不愿意花时间把事情做得更好。 | 1 | 2 | 3 | 4 | |
| 21 | 别人的感受对我来说并不重要。 | 1 | 2 | 3 | 4 | |
| 22 | 我向别人隐藏自己的情绪/情感。 | 1 | 2 | 3 | 4 | |
| 23 | 做任何事情我都很努力 | 1 | 2 | 3 | 4 | |
| 24 | 我会做些让别人感觉愉快的事。 | 1 | 2 | 3 | | 4 |
